# The Impact of the Combined Effect of Inhalation Anesthetics and Iron Dextran on Rats’ Systemic Toxicity

**DOI:** 10.3390/ijms25126323

**Published:** 2024-06-07

**Authors:** Dyana Odeh, Nada Oršolić, Emanuela Adrović, Nina Bilandžić, Marija Sedak, Irena Žarković, Nikola Lesar, Vedran Balta

**Affiliations:** 1 Division of Animal Physiology, Faculty of Science, University of Zagreb, Rooseveltov trg 6, 10000 Zagreb, Croatia; 2 Laboratory for Determination of Residues, Croatian Veterinary Institute, Savska cesta 143, 10000 Zagreb, Croatia; 3 Laboratory for Analysis of Veterinary Medicinal Products, Croatian Veterinary Institute, Savska cesta 143, 10000 Zagreb, Croatia

**Keywords:** Isoflurane, Sevoflurane, iron dextran, metal systemic toxicity, anesthetic systemic toxicity, toxic metals, essential metals

## Abstract

Disruption of any stage of iron homeostasis, including uptake, utilization, efflux, and storage, can cause progressive damage to peripheral organs. The health hazards associated with occupational exposure to inhalation anesthetics (IA) in combination with chronic iron overload are not well documented. This study aimed to investigate changes in the concentration of essential metals in the peripheral organs of rats after iron overload in combination with IA. The aim was also to determine how iron overload in combination with IA affects tissue metal homeostasis, hepcidin–ferritin levels, and MMP levels according to physiological, functional, and tissue features. According to the obtained results, iron accumulation was most pronounced in the liver (19×), spleen (6.7×), lungs (3.1×), and kidneys (2.5×) compared to control. Iron accumulation is associated with elevated heavy metal levels and impaired essential metal concentrations due to oxidative stress (OS). Notably, the use of IA increases the iron overload toxicity, especially after Isoflurane exposure. The results show that the regulation of iron homeostasis is based on the interaction of hepcidin, ferritin, and other proteins regulated by inflammation, OS, free iron levels, erythropoiesis, and hypoxia. Long-term exposure to IA and iron leads to the development of numerous adaptation mechanisms in response to toxicity, OS, and inflammation. These adaptive mechanisms of iron regulation lead to the inhibition of MMP activity and reduction of oxidative stress, protecting the organism from possible damage.

## 1. Introduction

**Figure 1:**
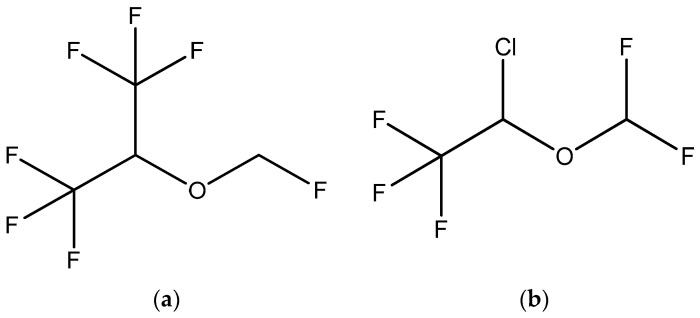
Chemical structure of Sevoflurane (**a**) and Isoflurane (**b**).

Widely used inhalation anesthetics, such as Sevoflurane, Isoflurane, and Desflurane, are small lipid-soluble molecules that significantly differ in terms of toxicity largely due to the relationship between their metabolism and toxicity development. Inhalation anesthetics are poorly metabolized and are mostly eliminated from the body via the respiratory system in an almost unchanged form. A smaller fraction is metabolized in the liver by the cytochrome oxidase P450 family and excreted by the kidneys [1]. Regardless of their metabolism to fewer toxic derivatives, sometimes inert compounds can be converted into reactive metabolites, such as reactive free radicals that engage in reactions with macromolecules, leading to the formation of toxic compounds and subsequent biochemical, structural, and functional disorders [2,3,4]. Accordingly, these biometabolites represent significant challenges in toxicology since they cause acute and chronic toxicity [5]. Inhalational anesthetics are associated with both neurotoxic effects and potential neuroprotective properties. On the other hand, nephrotoxicity and genotoxicity of inhaled anesthetics primarily arise from their metabolites, where the main targets are the liver, kidneys, and spleen. It is important to note that the effect of anesthetics depends on age and gender and that special attention should be paid to the disorder or disease from which the individual is suffering before the anesthetic is administered. For example, in elderly female rats, Sevoflurane causes more damage, but the level of damage is lower than with some other inhalation anesthetics, such as Isoflurane. Due to the slow metabolism of Isoflurane, very small amounts of inorganic fluoride are produced, so when examining its nephrotoxicity, a large number of studies show contradictory results [6,7]. Although Sevoflurane has not undergone formal testing, it has been approved for clinical use by the US Food and Drug Administration (FDA), probably due to the absence of carcinogenicity associated with the group of inhalation agents currently in use. For this reason, the evaluation of its toxicity as well as protection, both in vitro and in vivo, is being investigated today [5]. Therefore, one of the goals of this work is to evaluate the toxicity of Sevoflurane and Isoflurane with regard to long-term anesthetic exposure (Figure 1).

Also, human exposure to heavy metals represents a global public health concern [8]. Various definitions have been proposed for heavy metals, some based on density, some on atomic number or atomic weight, and some on chemical properties or toxicity. Based on toxicity, heavy metals are divided into two groups: (1) essential metals, which are less harmful at low concentrations (iron, copper, zinc, and cobalt), and (2) non-essential metals, which are harmful even at very low concentrations and lack known biological functions (aluminum, cadmium, mercury, lead, nickel, etc.) [9,10]. Essential heavy metals, such as iron, are necessary for various biological processes because they enable normal biological functions of cells, but in excessive amounts, they become harmful [10,11]. Metal ion homeostasis, maintained through tightly regulated mechanisms of uptake, storage, and excretion, is essential for life and is maintained within strict limits. Metal ion transporters participate in maintaining the necessary levels of various metal ions in cellular compartments [12]. Likewise, essential metals are important cofactors of several key enzymes and play an important role in various oxidation-reduction reactions [13]. Bioaccumulation of heavy metals leads to a series of acute and chronic toxic effects on various body tissues and organs [13]. Heavy metal toxicity primarily involves damage to the liver (hepatotoxicity), central nervous system (neurotoxicity), DNA (genotoxicity), and kidney (nephrotoxicity) in animals and humans [14]. The nature and severity of toxicity vary depending on the type and form of the heavy metal, the route and level of exposure, the chemical and valence state (inorganic vs. organic), the mode of exposure (acute vs. chronic), and demographic factors such as the individual’s age, sex, genetics, and overall susceptibility [11,15]. Simultaneous exposure to multiple metals can result in cumulative toxic effects, and high-dose heavy metal exposure can lead to severe complications, including abdominal pain, bloody diarrhea, and kidney failure [13]. If unrecognized or inadequately managed, the toxicity arising from metal exposure poses a clinically significant health issue, contributing to elevated morbidity and mortality rates [15].

Iron, despite its essential role, exhibits toxic properties when present in its free form. Therefore, conditions that lead to excessive iron accumulation and overload in body tissues can cause serious damage [16]. Its ability to mediate electron transfer can facilitate the production of reactive oxygen species (ROS), which are culpable for cellular and tissue injury. To mitigate these negative effects, iron is predominantly bound to proteins. In serum, it is mainly associated with transferrin, while intracellularly, it is activated by chaperones or stored within ferritin [17]. Therefore, the intracellular concentration of iron must be meticulously controlled due to the cytotoxicity of excess iron. Insufficient awareness regarding iron overload and its toxicological consequences often leads to tissue and organ damage. For example, anesthesiologists may not routinely include iron overload in preoperative assessments. Therefore, increased awareness of iron overload and its effects on anesthesia care is needed, particularly in at-risk patients [16]. The human body has several mechanisms to maintain iron homeostasis, including the controlled absorption, recycling, and storage of iron. All steps in maintaining iron homeostasis are strictly regulated at the systemic and cellular levels. The main regulator of iron homeostasis is hepcidin, a peptide hormone of 25 amino acids that is mostly produced in the liver but also in macrophages, pancreatic beta cells, kidneys, adipocytes, and lungs [18,19]. It restricts iron influx into plasma by inhibiting duodenal iron absorption, blocking the release of iron from macrophages that recycle senescent red blood cells, and curtailing the mobilization of stored iron from hepatocytes. This regulation of iron flow is achieved by the formation of a hepcidin–ferroportin complex, which causes the degradation of ferroportin (Fpn1), an iron transporter [20]. Therefore, hepcidin synthesis is upregulated in response to high iron levels, infection, and inflammation, while anemia, hypoxia, and erythropoiesis suppress its expression [21].

Very few articles show the effect of inhalation anesthetics on the concentration of zinc, copper, iron, manganese, and cobalt in the serum [16], but there are no data regarding the impact of inhalation anesthetics during conditions of iron excess, a key metal in numerous physiological and pathological processes, on changes in the concentration of essential and heavy metals in peripheral tissue. These observations motivated us to carry out the current work, which evaluates how iron overload affects iron uptake in the liver, kidney, spleen, and lung of rats after experimental iron overload, as well as subsequent alterations in the concentrations of essential and toxic metals in peripheral tissues. In addition, the aim was to determine how iron overload in combination with inhaled anesthetics affects metal homeostasis in tissues, hepcidin–ferritin levels, inflammation, and MMP levels considering physiological, functional features, and tissue barriers.

## 2. Results

### 2.1. Effect of Chronic Administration of Inhalation Anesthetics and Fe-Dextran on the Osmotic Fragility of Erythrocytes

The osmotic fragility assay of erythrocytes is a robust indicator of the degree of erythrocyte cell membrane damage caused by oxidative stress, altered enzyme synthesis or hemoglobin structural disturbances, and metabolic disorders within erythrocytes. In this study, we employed this method to assess the damage induced by chronic exposure to inhalation anesthetics and iron dextran.

**Figure 2:**
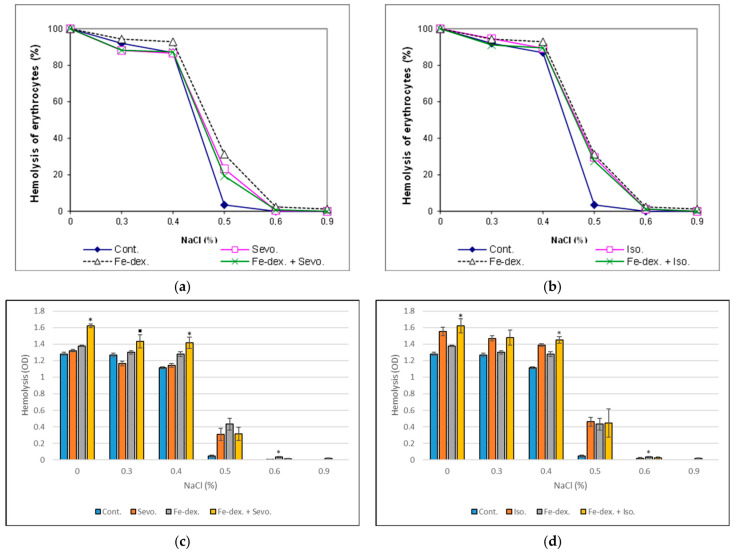
Percentage of osmotic fragility and statistical analysis of rat erythrocytes exposed to chronic administration of inhalation anesthetics—Sevoflurane (**a**,**c**), Isoflurane (**b**,**d**), iron dextran, and their combination. Rats (n = 10 per group) were treated with *ip* injection of iron dextran (50 mg/kg), exposed to 2.4 vol % Sevoflurane inhalation and 1.3 vol % Isoflurane inhalation, and exposed to a combination thereof where inhaled anesthetics were given 2 h after *ip* injection of iron dextran. Animals were treated every other day for 28 days. The results are expressed as the mean value of each experimental group ± SE. Statistically significant compared to the control group (* *p* < 0.05). ^■^ Statistically significant compared to the Sevoflurane group (^■^ *p* < 0.05). Abbreviations: *ip*, intraperitoneal; SE, standard error; Cont., control group; Sevo., Sevoflurane group; Iso., Isoflurane group; Fe-dex., iron dextran group; Fe-dex. + Sevo., group treated with iron dextran and Sevoflurane; Fe-dex. + Iso., group treated with iron dextran and Isoflurane.

Figure 2 shows that treatment with Fe-dextran and its combinations alters the membrane properties of erythrocytes, leading to increased osmotic fragility. In these experimental groups, 50% hemolysis occurred at a NaCl concentration of 0.50%, while in the control group, 50% hemolysis was observed at a NaCl concentration of 0.45%. Statistical analysis showed that there is a significant difference in erythrocyte fragility in the group treated with Fe-dextran + Sevoflurane at concentrations of 0.4 and 0.0% NaCl compared to the control group (Figure 2c). This same effect is visible in the group treated with Fe-dextran + Isoflurane, where a statistical difference exists at the same concentrations of NaCl compared to the control group (Figure 2d). Furthermore, in the group treated with Fe-dextran, a significant difference compared to the control (*p* < 0.05) exists at a NaCl concentration of 0.6%.

### 2.2. Concentrations of Essential Metals in Peripheral Tissues of Rats Treated with Chronic Administration of Inhalation Anesthetics and Fe-Dextran

Iron, as an essential metal, is important for cell metabolism, proliferation, and differentiation, and it is important for many physiological processes, including immunity, oxygen transport, electron transfer, and catalysis of numerous reactions [22]. On the other hand, iron is a bioactive element, and excess free iron is toxic to the body. To determine which tissues accumulate the most metals according to physiological, functional features, and tissue barriers, we assessed the levels of essential and toxic metals in peripheral tissues using the inductively coupled plasma mass spectrometry method (ICP-MS).

Table 1 and Table 2 show the accumulation levels of iron and other essential metals in the peripheral tissues of rats treated with chronic administration of inhalation anesthetics and Fe-dextran. As shown in Table 1, there is a significant accumulation of iron (mg/kg) in all peripheral organs in groups treated with Fe-dextran, Fe-dextran + Sevoflurane, and Fe-dextran + Isoflurane compared to the control group.

In the group exposed to Sevoflurane, the concentration of copper (mg/kg) was significantly decreased in the lungs (Table 2). In the Isoflurane-exposed group, copper concentration was significantly decreased in the liver, while zinc concentration was significantly increased in the liver, kidneys, and lungs (Table 2). In the group treated with Fe-dextran, zinc concentration was significantly increased in all peripheral organs, whereas manganese concentration was elevated in the liver and spleen, and copper concentration significantly decreased in the kidneys. Likewise, zinc concentration was significantly increased in all peripheral organs in the Fe-dextran + Sevoflurane group. Manganese concentrations were elevated in the liver, lungs, and spleen in the Fe-dextran + Sevoflurane group, while selenium concentration was decreased in the kidneys and lungs. Additionally, Table 2 shows an increase in copper concentration in the spleen tissue in the Fe-dextran + Sevoflurane group.

In the group treated with Fe-dextran + Isoflurane, the concentrations of manganese and copper were statistically significantly decreased in the liver and kidneys. Conversely, the concentration of manganese was increased in the lungs and spleen. Additionally, the concentration of copper in the lungs was decreased, whereas it was increased in the spleen. Likewise, the concentration of selenium was significantly decreased in the liver and kidneys but increased in the lungs and spleen in Fe-dextran + Isoflurane-treated group (see Table 2).

### 2.3. Toxic Metal Concentrations in Peripheral Tissues of Rats Treated with Chronic Administration of Inhalation Anesthetics and Fe-Dextran

The results of the analysis of toxic metals are presented in Table 3 and Table 4. The greatest changes in the accumulation of toxic metals in all peripheral tissues were observed in the group treated with Fe-dextran + Isoflurane. Aluminum concentration (Table 3) in the group exposed to Sevoflurane was statistically significantly increased in the spleen compared to the group exposed to Isoflurane (*p* < 0.01). In the Fe-dextran-treated group, aluminum concentration (mg/kg) was significantly increased in the liver and spleen. In the group treated with Fe-dextran + Sevoflurane, aluminum accumulation was observed in all peripheral organs except the spleen (Table 3). In the group treated with Fe-dextran + Isoflurane, aluminum concentration was significantly decreased in the kidneys and significantly increased in the lungs.

Table 4 shows the results of cadmium and lead (μg/kg), from which a significant decrease in cadmium levels in the liver, kidneys, and lungs was observed in the group exposed to Isoflurane. Lead concentration was significantly increased in the liver and spleen of the Fe-dextran-treated group compared to the group exposed to Isoflurane and the control group. In the Fe-dextran + Sevoflurane-treated group, lead concentration was elevated in the liver, kidneys, and lungs, while cadmium concentration was significantly decreased in the kidneys but increased in the lungs (Table 4). In the Fe-dextran + Isoflurane-treated group, the cadmium concentration was significantly decreased in the liver and increased in the spleen. Additionally, in the Fe-dextran + Isoflurane-treated group, the lead concentration was decreased in the kidneys and significantly increased in the lungs and spleen (Table 4).

### 2.4. Changes in the Hepcidin–Ferritin Level after Chronic Administration of Inhalation Anesthetics and Iron Dextran

Anesthesiologists do not routinely consider iron overload as part of the preoperative assessment. Therefore, there is a need for increased awareness of iron overload and its effects on anesthesia care, especially in high-risk patients, due to the potential for toxicity leading to tissue and organ damage. Consequently, for the stated reason, in this work, we wanted to assess the levels of hepcidin and ferritin despite the existence of a highly organized and regulated network that maintains iron homeostasis in the body. Likewise, serum ferritin, a known acute-phase protein, can be used as a marker for several inflammatory pathologies, especially in the context of chronic use of inhalation anesthetics and Fe-dextran.

**Figure 3:**
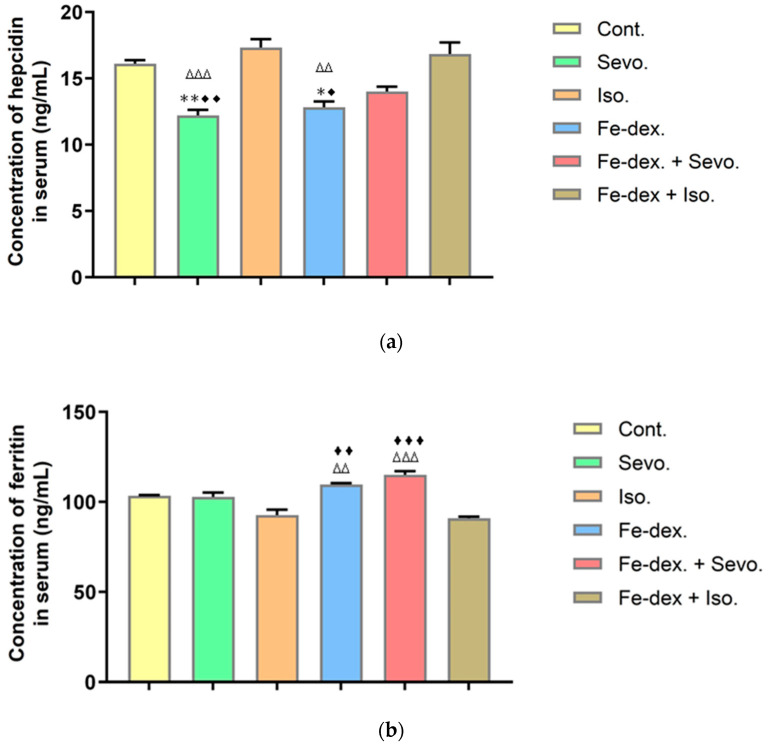
Concentration of hepcidin (**a**) and ferritin (**b**) in the serum of rats exposed to chronic administration of inhalation anesthetics and iron dextran and their combination. Rats (n = 10 per group) were treated with *ip* injection of iron dextran (50 mg/kg), exposed to 2.4 vol % Sevoflurane inhalation and 1.3 vol % Isoflurane inhalation, and exposed to a combination thereof where inhaled anesthetics were given 2 h after *ip* injection of iron dextran. Animals were treated every other day for 28 days. The results are expressed as the mean value of each experimental group ± SE. * Statistically significant compared to the control group (* *p* < 0.05; ** *p* < 0.01); ^∆^ statistically significant compared to the Isoflurane group (^∆∆^ *p* < 0.01; ^∆∆∆^ *p* < 0.001); ^♦^ statistically significant compared to the Fe-dex. + Iso.-treated group (^♦^ *p* < 0.05; ^♦♦^ *p* < 0.01; ^♦♦♦^ *p* < 0.001). Abbreviations: *ip*, intraperitoneal; SE, standard error; Cont., control group; Sevo., Sevoflurane group; Iso., Isoflurane group; Fe-dex., iron dextran group; Fe-dex. + Sevo., group treated with iron dextran and Sevoflurane; Fe-dex. + Iso., group treated with iron dextran and Isoflurane.

From the serum of rats treated with chronic administration of inhalation anesthetics and iron dextran and their combination, it can be seen from Figure 3a that the concentration of hepcidin was statistically significantly reduced in the groups treated with Sevoflurane (reduced by approximately 1.4×) and Fe-dextran (reduced by approximately 1.3×) compared to groups treated with Isoflurane or Fe-dextran + Isoflurane and the control group.

Figure 3b shows a significantly increased concentration of ferritin in the groups treated with Fe-dextran and Fe-dextran + Sevoflurane compared to the groups treated with Isoflurane (*p* < 0.01; *p* < 0.001) and Fe-dextran + Isoflurane (*p* < 0.01; *p* < 0.001).

### 2.5. MMP-2 and MMP-9 Concentrations in Rat Serum after Chronic Administration of Inhalation Anesthetics and Iron Dextran

Matrix metalloproteinases (MMPs) have been proposed as markers of many pathological conditions due to their ability to degrade extracellular matrix components and tissue remodeling. Although MMP activity is essential in numerous cell biological processes and fundamental physiological events such as tissue remodeling, angiogenesis, bone development, wound healing, and breast involution, the growing interest in MMP function mainly stems from their role in several pathological conditions, particularly chronic inflammatory diseases. In this study, given the attention to chronic inflammation, tissue damage, and metal accumulation, we aimed to evaluate the serum levels of MMP-2 and MMP-9 in rats.

**Figure 4:**
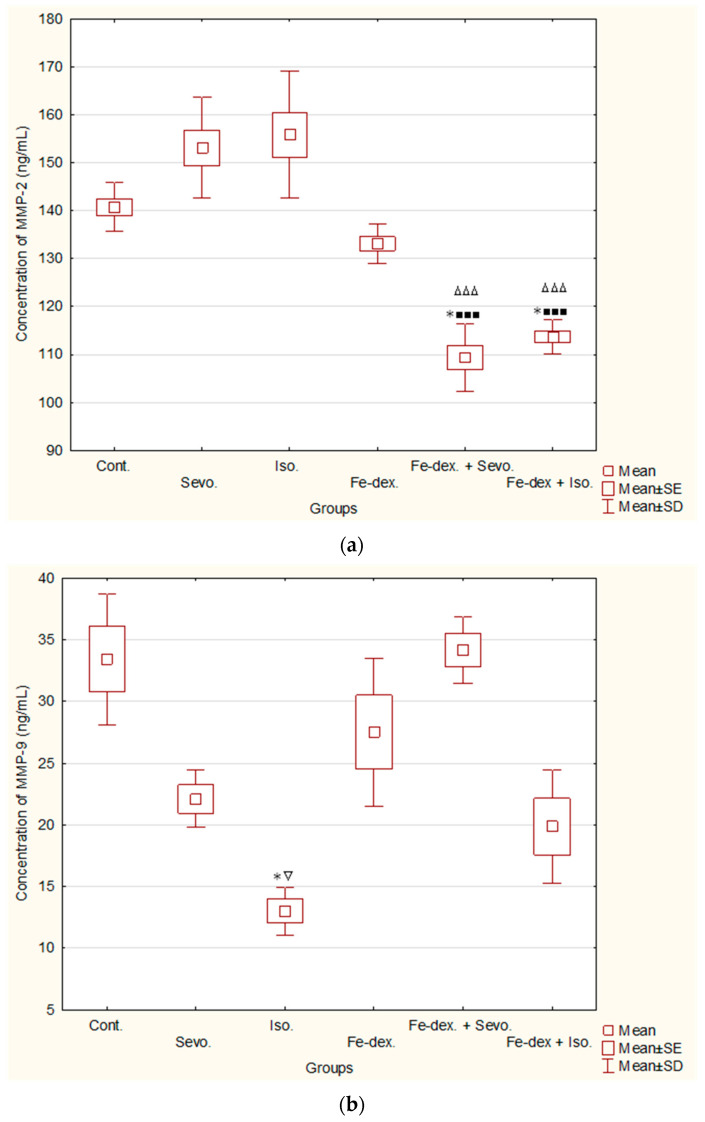
Concentrations of MMP-2 (**a**) and MMP-9 (**b**) in the serum of rats exposed to chronic administration of inhalation anesthetics and iron dextran and their combination. Rats (n = 10 per group) were treated with *ip* injection of iron dextran (50 mg/kg), exposed to 2.4 vol % Sevoflurane inhalation and 1.3 vol % Isoflurane inhalation, and exposed to a combination thereof where inhaled anesthetics were given 2 h after *ip* injection of iron dextran. Animals were treated every other day for 28 days. The results are expressed as the mean value of each experimental group ± SE. * Statistically significant compared to the control group (* *p* < 0.05); ^■^ statistically significant compared to the Sevoflurane group (^■■■^ *p* < 0.001); ^∆^ statistically significant compared to the Isoflurane group (^∆∆∆^ *p* < 0.001); ^∇^ statistically significant compared to the Fe-dex. + Sevo.-treated group (^∇^ *p* < 0.05). Abbreviations: *ip*, intraperitoneal; SE, standard error; Cont., control group; Sevo., Sevoflurane group; Iso., Isoflurane group; Fe-dex., iron dextran group; Fe-dex. + Sevo., group treated with iron dextran and Sevoflurane; Fe-dex. + Iso., group treated with iron dextran and Isoflurane.

The results of the serum concentration of MMP-2 and MMP-9 following chronic administration of inhalation anesthetics and iron dextran and their combination are shown in Figure 4. Figure 4a demonstrates a statistically significant decrease in the concentration of MMP-2 in the group treated with Fe-dextran + Sevoflurane compared to the groups exposed to Sevoflurane (*p* < 0.001), Isoflurane (*p* < 0.001) and the control group (*p* < 0.05). Similarly, MMP-2 concentration was significantly reduced in the Fe-dextran + Isoflurane-treated group compared to the groups exposed to Sevoflurane (*p* < 0.001), Isoflurane (*p* < 0.001) and the control group (*p* < 0.05).

The concentration of MMP-9 (Figure 4b) was statistically significantly decreased in the group exposed to Isoflurane (13.02 ± 0.97) compared to the group treated with Fe-dextran + Sevoflurane (34.15 ± 1.34; *p* < 0.05) and the control group (33.42 ± 2.66; *p* < 0.05).

## 3. Discussion

Iron is a key element for the functioning of all cells, with its most important functions being the oxygen transport to all cells, DNA synthesis, and the regulation of cell growth and differentiation. Additionally, iron serves as an important cofactor for various enzymes and proteins involved in numerous biochemical reactions. Iron deficiency is very common and can lead to conditions such as anemia, so it is usually used for a long time in the treatment of numerous anemias and other diseases caused by iron deficiency [23]. Some iron-deficiency anemias can increase the risk of complications affecting the heart, liver, kidney, or lungs. Despite the tight regulation of iron levels by hepcidin, disruptions in iron homeostasis frequently occur, leading to the release of free or labile iron. This free iron leaves serious consequences due to toxicity and increased oxidative stress [24]. Iron overload causes specific cell damage that depends on the protective ability of cells and on the different sensitivity of tissues. Iron exerts its toxicity through a series of reactions with reactive oxygen species (ROS), generating highly toxic hydroxyl radicals (OH). These radicals cause lipid peroxidation, amino acid oxidation, and, consequently, protein fragmentation, disruption of protein–protein interactions, as well as DNA damage. This cascade ultimately leads to damage, the impairment of cellular mechanisms, and damage to organs and tissues [23]. It has also been confirmed that long-term exposure to anesthesia leads to neurodegenerative changes and a decline in cognitive abilities by disrupting iron homeostasis through its excessive accumulation in the hippocampus [25]. Therefore, this study investigated the effects of prolonged iron administration alone and in combination with inhalation anesthetics, whose long-term exposure causes damage through increased production of toxic metabolites and reactive radicals.

Although the toxicity of inhalation anesthetics has been mostly investigated in the brain, there are no data regarding their impact on peripheral organs. Therefore, the purpose of this study was to investigate the uptake of iron in the liver, kidney, spleen, and lungs of rats following experimental iron overload, as well as its influence on the concentration of essential and toxic metals in peripheral tissues. In addition, the aim was to examine how iron overload in combination with inhalation anesthetics affects metal homeostasis in tissues, the interrelationship between hepcidin and ferritin levels, inflammation, and MMP levels according to physiological, functional features, and tissue barriers.

According to the obtained results, an increased iron accumulation in the liver, kidneys, spleen, and lungs was observable in the group treated with iron dextran (Table 1). The highest accumulation of iron compared to the control group is visible in the liver (~19-fold higher), followed by the spleen (~6.7-fold higher), lungs (~3.1-fold higher), and kidneys (~2.5-fold higher). The liver emerges as a major target of iron toxicity due to its central role in the metabolism, circulation, and storage of this metal through macrophages of the reticuloendothelial system. It seems that the number of macrophages in the reticuloendothelial system appears to be crucial for the capture and accumulation of iron in the liver and spleen (Table 1). In addition to the macrophages of the reticuloendothelial system, blood flow is also important, as well as the gradual sequence of metal mobilization from the liver to the kidney as an excretory organ. The kidneys, characterized by a faster metabolic rate than other organs, exhibit a significantly lower rate of iron accumulation despite their role in the excretion and regulation of metal homeostasis.

Furthermore, according to our results, there is a clear correlation between the increased iron concentration in the liver, spleen, lungs, and kidneys with the increased levels of manganese and zinc, while the copper levels remain unchanged or decreased (Table 2). The most significant reductions in copper are observed in the combination of Fe-dextran with Isoflurane in the liver, lungs, and kidneys (Table 2). Some experimental and clinical studies in animals and humans have also indicated an interaction between iron and zinc during absorption from the small intestine. According to Vayenas et al. [26], intracellular iron loading results in intracellular accumulation of zinc that is stored in the liver bound to metallothionein and/or ferritin. Moreover, parenteral administration of iron resulted in an increased concentration of manganese in the liver and spleen. Likewise, according to Ye and Kim [27], iron overload facilitates manganese deposition. One potential mechanism is that manganese may act as an antioxidant against iron-induced oxidative stress in peripheral tissues. Manganese, as an essential transition metal, plays an important role as a cofactor for various metabolic and antioxidant enzymes and is involved in blood sugar regulation, bone growth, blood clotting, and the immune system. It is possible that manganese and iron interact synergistically during the transfer from the plasma to the brain and other organs.

On the other hand, exposure of rats to anesthetics in the presence of iron results in increased iron accumulation across all tissues (Table 1). Iron overload in the liver and spleen after Isoflurane exposure leads to increased cell death via ferroptosis [28], as evidenced by changes in organ weight [29] and a significant reduction in essential metals in a state of iron-induced oxidative stress (Table 2). Another potential explanation involves increased oxidative stress after ischemia–reperfusion conditions, given that Isoflurane causes congestion, respiratory restriction, and asphyxiation. These data suggest that Isoflurane increases toxicity and may induce enhanced destruction of red blood cells after inflammation. Moreover, it is possible that reduced metabolism and enzymatic activity after anesthetic administration may disrupt the processes of metal transfer and removal, while increased oxidative stress in the spleen and liver leads to increased erythrocytes breakdown and iron loss. The presence of excess free iron released from the heme breakdown by heme oxygenase-1 (HO-1), which is ubiquitously abundant in reticuloendothelial organs, contributes to liver damage and spleen dysfunction [30,31]. HO-1 activity is coupled with Fe-ATPase and iron mobilization. Its activity in the spleen, as a hematopoietic and lymphatic organ, is approximately 20-fold higher compared to other tissues [32]. Increased HO-1 activity in the spleen degrades heme, releasing iron for transport into the lumen of the endoplasmic reticulum by Fe-ATPase. Furthermore, Fe-ATPases facilitate the exocytosis of free iron, thereby protecting the cells. The protective effects of HO-1, both as an antioxidant and anti-inflammatory molecule, have been confirmed in response to a number of different stress stimuli, including various pro-oxidants and pro-inflammatory mediators [33]. The induction of HO-1 is a general adaptive cellular mechanism that provides protection against damage under stress conditions, and HO-1 upregulation can be achieved through the activation of multiple different regulatory mechanisms.

The increased accumulation of manganese, copper, zinc, and selenium in the spleen across almost all experimental groups (Table 2) suggests an upregulation of antioxidant enzyme synthesis in response to oxidative stress. This response is primarily mediated by HO-1, which is largely under the control of the redox-sensitive transcription factor nuclear factor erythroid 2-related factor 2 (Nrf2). Nrf2 binds to the antioxidant response element (ARE) in the promoter region of various antioxidant genes. Although iron is toxic, its release can induce oxidative stress and affect HO-1 translocation into the nucleus, leading to damage to erythrocytes in the red pulp of the spleen. The exported iron is transported via plasma transferrin into the systemic circulation, where it is utilized by specialized bone marrow macrophages for the synthesis of hemoglobin in erythrocytes during erythropoiesis. Therefore, one possible explanation for the reduced iron levels in the spleen of the groups treated with Fe-dextran in combination with Isoflurane (Table 2) is related to the increased breakdown and subsequent need for erythrocytes and hemoglobin synthesis due to reduced level of oxygen in the body. This assertion is supported by the observed increase in the number of erythrocytes and hemoglobin in these treated groups, as indicated by the increase in the number of erythrocytes and hemoglobin in these treated groups [29].

Moreover, we observed a decreased level of copper in the lungs of rats exposed to inhalation anesthetics (Sevoflurane or Isoflurane), which indicates increased oxidative stress and insufficient synthesis of antioxidant enzymes, given that copper is essential for the activity of Cu/Zn-superoxide dismutase (Table 2). Copper is an essential trace element required for various physiological processes in the body, including antioxidant defense, energy production, and connective tissue formation. Also, reduced copper levels can affect the immune system, as copper is involved in immune cell function and the inflammatory response. Copper deficiency could potentially compromise the lung’s ability to defend against inflammation, which is relevant to our study where chronic inflammation is present. Chronic exposure to Sevoflurane demonstrated fewer toxic effects compared to Isoflurane, which showed toxicity in all examined tissues [34]. Thus, the presence of iron following exposure to Isoflurane predominantly affects the liver, spleen, lungs, and kidneys. Isoflurane exposure is associated with reduced lung function and death of type II alveolar cells through increased lactate production, as well as reduced surfactant levels due to inhibition of phosphatidylcholine synthesis. In addition, Isoflurane causes a decrease in the function of alveolar type II cell Na/K-ATPase, which regulates epithelial barrier function and edema clearance during lung damage [35,36,37].

It is known that toxic metals such as lead and aluminum are known to cross the blood–brain barrier and accumulate in the brain. Our data show that elevated iron levels correlate with increased concentrations of aluminum, lead, and cadmium in the liver, kidneys, lungs, and spleen in almost all experimental groups (Table 3 and Table 4). However, it is interesting that the combination of iron with Sevoflurane (Fe-dextran + Sevoflurane) leads to the highest accumulation of aluminum in all organs except the spleen. In contrast, the combination of Fe-dextran + Isoflurane results in increased aluminum levels only in the lungs, not in other tissues (Table 3). The combination of Fe-dextran and Sevoflurane appears to significantly elevate iron and essential metal levels in the liver, kidney, lung, and spleen compared with Isoflurane. Possibly, this increase in essential metals may represent a homeostatic mechanism to mitigate the toxicity caused by heavy metals and anesthetic metabolites. Also, Sevoflurane may differentially affect numerous proteins and channels critical to iron metabolism, warranting further investigation. Iron overload in the liver, coupled with the accumulation of other toxic metals, such as aluminum and lead, induces oxidative stress, reduces the liver’s antioxidant capacity, impairs hepatocyte function [29,31], and can result in fibrosis, cirrhosis, or the development of hepatocellular carcinoma [38]. Thus, intraperitoneal injection of iron initially leads to phagocytosis by peritoneal macrophages [39]. Iron subsequently accumulates in tissue macrophages of the liver, spleen, bone marrow, and other organs (siderosis phase). Once the capacity of this system is exceeded, iron begins to deposit in the parenchymal cells of organs, such as the liver, heart, pancreas, and other endocrine glands, leading to damage and dysfunction. Likewise, this accumulation of iron in the body is associated with increased oxidative stress, as demonstrated in our previous work [29]. The accumulation of essential metals in the mentioned tissues indicates the upregulation of antioxidant enzyme synthesis in response to oxidative stress, which was particularly evident in all tissues (Table 2). Elevated oxidative stress and the production of highly reactive cytotoxic hydroxyl radicals (·HO) are facilitated by iron, which catalyzes the interaction of superoxide (O_2_^·−^) and hydrogen peroxide (H_2_O_2_) via Fenton or Haber–Weiss reactions. Consequently, iron overload is linked to cardiovascular and chronic kidney diseases characterized by iron deposition in the glomerulus, proximal and distal tubules, glomerulosclerosis, tubular atrophy, and interstitial fibrosis [40,41].

The reduced availability of nitric oxide (NO) in the liver and kidneys, confirmed in our previous work [29], is a significant factor contributing to increased vascular and subsequent damage to other tissues [42]. However, the literature on kidney or liver damage due to iron overload and exposure to anesthetics is highly contradictory. Some studies report significant damage, while others indicate an absence of expected damage. Our data reveal minimal biochemical changes in kidney and liver parameters, except in the groups treated with Fe-dextran + Isoflurane, where the damage was greater. This suggests that the body employs multiple regulatory mechanisms to maintain iron homeostasis, and various factors can influence organ injury. These factors include the dose and duration of iron intake, sex, age, animal species, iron formulation, and various adaptive mechanisms such as HO-1 induction. Kidney damage, in particular, appears to be significantly influenced by the iron formulation used [43].

The binding of iron by transferrin in the circulation and by ferritin within cells ensures the safe transport and storage of this essential but highly toxic element. This protective mechanism, evident in some groups, is shown in Figure 3a, where we confirmed that increased iron levels are accompanied by elevated ferritin levels (Figure 3b), while hepcidin levels are decreased (Figure 3a). Elevated ferritin levels act as a protective mechanism against the harmful effects of free iron by storing and sequestering excess iron in a non-toxic form. According to Kell and Pretorius [44], serum ferritin is an important inflammatory marker associated with damaged cells, hydroxyl radical levels, and various inflammatory and degenerative diseases. In our study, ferritin levels were approximately 20% higher in groups treated with Fe-dextran and Fe-dextran + Sevoflurane (Figure 3b), whereas hepcidin levels were increased in the groups exposed to Isoflurane and Fe-dextran + Isoflurane (Figure 3a). Hepcidin, a key regulator of iron homeostasis, influences iron metabolism by regulating intestinal iron absorption, recycling iron from macrophages, and controlling hepatic iron storage. According to the literature [45,46], hepcidin levels should be increased during iron overload, inflammation, or hypoxia. We believe that hypoxia is one of the reasons for the increase in hepcidin levels in the groups exposed to Isoflurane and Fe-dextran + Isoflurane. It is interesting that hepcidin levels increase during repeated iron administration for up to two weeks, but the chronic treatment of rats with iron does not lead to changes in hepcidin levels, even at higher doses [47]. These findings are consistent with our data, where we anticipated significant changes in hepcidin levels. It appears that the organism’s adaptive and regulatory abilities in response to excessive iron intake are more robust than expected, suggesting that hepcidin is not the sole regulator of iron homeostasis but that numerous liver proteins and environmental conditions also contribute to these adaptative mechanisms [48].

Ferritin level correlates with changes in erythrocyte morphology, iron storage, and hematological changes [29]. Toxicity, the presence of inflammation, and oxidative stress are associated with alterations in erythrocyte morphology through the oxidation of membrane and cytoskeletal proteins. Oxidative stress modifies the structure and function of the erythrocyte membrane, leading to increased osmotic fragility and decreased cell fluidity. The osmotic resistance of erythrocytes is an excellent indicator of the presence of toxic metabolites that disturb the osmotic integrity of cells. Increased hemolysis is evident in all treated groups, where 20–30% of lysed erythrocytes were observed in a 0.5% NaCl solution (Figure 2). Erythrocyte lysis results from oxidative damage to the erythrocyte membrane, reducing fluidity and the ability to resist osmotic changes [49]. It should be emphasized that heme iron is also a potent pro-oxidant biomolecule capable of initiating cellular signaling events, as ROS generated by its reaction can act as a secondary messenger. Thus, elevated levels of H_2_O_2_, produced due to the pro-oxidative effect of heme iron, have been shown to enhance the activity of protein kinase C (PKC) and Na/K-ATPase. In erythrocytes, iron overload stimulates Na/K-ATPase activity, potentially through direct interaction with the enzyme or modification of the lipid microenvironment. According to Sousa et al. [50], iron overload leads to changes in membrane elasticity, hindering the passage of erythrocytes through narrow capillaries and affecting the lateral mobility of Na/K-ATPase, both of which are crucial for cell function. Na, K-ATPase is a key protein regulating the cellular volume of erythrocytes. Iron overload can increase the risk of infection and result in a weakened immune system cell response due to shortened lifespan of immune cells, reduced phagocytic activity, or death of polymorphonuclear leukocytes and monocytes [40,41,51].

Such changes result in alterations in cytokine production by macrophages as well as changes in their function. Possibly, the reduced function of macrophages and neutrophils, the primary producers of MMP-2 and MMP-9, along with elevated ferritin levels, appear to be the key factors contributing to the minor changes in MMP levels observed in our results (Figure 4). These findings are consistent with Otogawa et al. [52], who demonstrated that in a model of acute and chronic liver injury, the expression of MCP-1, IL-6, TNF, and MMPs was unchanged in rats compared to those on an iron-deficient diet. According to Wang et al. [53], low intracellular iron levels in immune cells, including macrophages, selectively modulate the TLR4-activated inflammatory response, resulting in reduced LPS-induced cytokine expression. This suggests that an iron-dependent mechanism modulates the TLR signaling pathway, and manipulation of iron homeostasis could represent a novel strategy to control inflammation. Controversially, iron deficiency has been shown to impair the intra-hepatic lymphocyte-mediated immune response [54]. The inflammatory process and the accumulation of neutrophils and macrophages in damaged tissue are crucial for tissue regeneration after iron-induced damage. Thus, inflammatory cells in the liver are essential for removing necrotic cells after intense hepatocyte necrosis. In addition to cell clearance, these inflammatory cells stimulate the division of healthy cells around the necrotic area, which is crucial for successful tissue regeneration. The reduced level of MMP-2 observed in the combination of Fe-dextran with inhalation anesthetics (Figure 4a) may partially reflect the anti-inflammatory effects of anesthetics, as well as the immunosuppressive effects of heavy metals [55,56,57]. The decreased MMP-2 levels in these groups indicate mechanisms of adaptation to induced toxicity. Overall, the slight changes in MMP-9 and MMP-2 levels (Figure 4) across all treated groups contribute to a protective mechanism. Although iron is considered crucial in regulating the immune response, it is noteworthy that iron overload did not significantly increase myeloperoxidase levels in the liver, lungs, small intestine, or colon in sucrose-induced iron overload rat models [47]. In contrast, other studies suggest that iron can exacerbate the inflammatory status of inflammatory bowel disease in a rat model, especially due to its effect on the microbial composition, which is critically important in this disease. It has been confirmed that oral iron supplementation can disrupt intestinal bacterial diversity, leading to changes in disease activity, as some bacteria are more dependent on iron than others [58,59].

Our data indicate that environmental conditions influence the plasticity of macrophages and the relationship between the M1/M2 macrophage phenotypes. Activation of the HO-1 enzyme is closely associated with substances that promote the polarization of macrophages towards the M2 anti-inflammatory phenotype, such as IL-10. Although we did not measure HO-1 levels, its activity is critical in the oxidative breakdown of heme into three by-products: biliverdin (subsequently converted into the antioxidant and anti-inflammatory bilirubin), ‘free’ iron, and carbon monoxide (CO) [60]. Despite being a toxic gas, CO can have beneficial effects [61,62,63]. HO-1 contributes to anti-inflammatory activity and promotes polarization towards the M2 phenotype [64,65], while iron is necessary for the constitutive degradation of the hypoxia-inducible factor (HIF). Iron deficiency in macrophages could increase HIF activation [66] but also prevent the generation of functional iron-containing enzymes such as lipoxygenase, prostaglandin H synthase, and myeloperoxidase, which are important in arachidonate metabolism. Iron also influences the synthesis of the enzyme tyrosine hydroxylase, which limits the rate of catecholamine biosynthesis. Catecholamines produced in macrophages have the ability to enhance inflammatory responses [67]. Therefore, iron appears to regulate macrophage function through multiple pathways, affecting their polarization, enzymatic activity, and overall role in inflammation and immune responses.

These animal models do not mimic situations in patients receiving these anesthetics. Some evidence suggests that Isoflurane metabolism to trifluoroacetic acid and inorganic fluoride by human liver microsomes in vitro is catalyzed by cytochrome P450 2E1 (CYP2E1). According to Kharasch et al. [68], P450 2E1 is the predominant P450 isoform responsible for human clinical Isoflurane metabolism in vivo. In addition, chronic ethanol intake also induces CYP2E1. Thus, iron-induced liver damage in mice does not fully correspond to damage in humans who consume large amounts of alcohol for a long time due to possible interactions of iron in ethanol-induced liver damage and increased induction of CYP2E1. CYP2E1 may constitute the primary source of superoxide anion production, leading to the formation of hydroxyl radicals via an iron-catalyzed Haber–Weiss reaction, indicating a potential role for iron in CYP2E1-induced free radical production [69,70]. Also, hypersensitivity reactions should be highlighted as idiosyncratic drug-induced hepatitis (IDDIH) resulting from the production of trifluoroacetylated (TFA) liver proteins during the metabolism of volatile anesthetics, mostly halothane, by CYP2E1. These proteins, acting as neoantigens, stimulate the formation of autoantibodies against liver tissue. Autoantibodies that react with CYP2E1 are significantly elevated in patients with halothane hepatitis (45–70%) [71], especially IgG4 and 58 kDa endoplasmic reticulum protein autoantibodies (ERp58) [72].

In general, obtained data indicate that long-term administration of iron leads to its accumulation in all organs, particularly in the liver and spleen. Elevated iron levels are associated with the accumulation of heavy metals in tissues, contributing to increased oxidative stress and hemolysis of erythrocytes. Increased oxidative stress disrupts the balance of essential metals in all tissues, which indicates the need for increased synthesis of antioxidant enzymes. Furthermore, the use of inhalation anesthetics exacerbates iron toxicity, with Isoflurane exposure showing the most pronounced effects. Our results suggest that hepcidin and ferritin are regulated by a complex interplay of chronic inflammation, oxidative stress, reorganization of damaged tissues, and free iron levels. Furthermore, it is evident that hepcidin and ferritin are not the sole proteins involved in iron regulation. Numerous liver proteins, enzymes, and their products, as well as environmental conditions, affect various signaling pathways and regulate numerous adaptation mechanisms. This model, even if it does not fully correspond to human conditions, still provides a foundation for future studies aimed at elucidating the molecular mechanisms of dextran-induced iron overload, as well as understanding the consequences of iron dysregulation in humans and animals exposed to inhalation anesthetics.

## 4. Materials and Methods

### 4.1. Chemicals

Iron dextran from Santa Cruz Biotechnology (Dallas, TX, USA) was used in this study as heavy metal, while Sevoflurane and Isoflurane from Baxter (Deerfield, IL, USA) were used as inhalation anesthetics. Additionally, saline solution or 0.9% sodium chloride manufactured by B. Braun Adria d.o.o. (Zagreb, Croatia) was used in the experimental treatment of animals.

### 4.2. Iron Dextran Solution

A solution of iron dextran was prepared by dissolving FeH_2_O_4_S in a subchronic dose of 50 mg/kg in purified water (*aqua pro*) produced by B. Braun Adria d.o.o., Croatia.

### 4.3. Animals and Ethics Statement

Both sexes of inbred Y59 rats (3 months old) obtained from the Department of Animal Physiology, Faculty of Science, were used in this study. This study was performed on a total of 60 Y59 rats randomly divided into 6 groups of 10 animals, each according to the treatment. Animals had access to food (standard diet GLP, 4RF21, Mucedola, Settimo Milanese MI, Milano, Italy) and water ad libitum, and housing conditions were standard.

The Ethical Committee of Faculty of Science, University of Zagreb, approved this research (approval code: 251-58-10617-19-57; date of approval: 23 January 2019). The research was performed in accordance with ethical and legal principles valid in the Republic of Croatia (Law on Amendments to the Law on Animal Welfare, NN 37/13 [73]; Law on Animal Welfare, NN 102/2017 [74]; Regulation on the Protection of Animals Used for Scientific Purposes, NN 55/13 [75]) and according to the Guide for the Care and Use of Laboratory Animals, DHHS (NIH) Publ # 86-23, National Research Council [76].

### 4.4. Experimental Design

Experimental design and dose selection are described in detail in our paper [29]. Briefly, animals were individually labeled and weighed before the start of the experiment, as well as during the experiment and on the day of sacrifice. The experimental animals were divided into 6 groups of 10 animals. The first group served as a control group and received 0.9% NaCl every other day during the treatment. The second group of animals was exposed for two hours to 2.4 vol % inhaled anesthetic Sevoflurane during the treatment. The third group was exposed for two hours to 1.3 vol % inhaled anesthetic Isoflurane, and the fourth group received intraperitoneal (*ip*) injection of iron dextran solution in a subchronic dose of 50 mg/kg. The fifth group of animals received 2.4 vol % inhaled anesthetic Sevoflurane two hours after *ip* injection of iron dextran solution (50 mg/kg), while the sixth group of animals received 1.3 vol % Isoflurane inhaled anesthetic two hours after *ip* injection of iron dextran solution (50 mg/kg) during the treatment. The animals were treated every other day for 28 days, and immediately after the last treatment, the animals were anesthetized and analgesic *ip* with a combination of xylazine (Xylapan^®^, Bioveta, Ivanovice na Hané, Czech Republic) at a dose of 10 mg/kg, and ketamine (Narketan^®^, Bioveta, Ivanovice na Hané, Czech Republic) at a dose of 75 mg/kg and sacrificed to collect the blood, and organs for further analysis.

### 4.5. Blood Sampling

Blood samples were collected from the abdominal aorta (*A. abdominalis*) according to the guidelines of the Clinical Laboratory Standards Institute [77] and the World Health Organization [78]. Thus, collected blood was used for spectrophotometric analyses.

### 4.6. The Osmotic Fragility Test of Erythrocytes

A series of tubes with 0.9 mL of sodium chloride solution (0.9%, 0.8%, 0.7%, 0.6%, 0.5%, 0.4%, 0.3%, 0.2%, 0.1%) was prepared. A total of 0.9 mL of distilled water (0%) was added to one test tube. At the same time, 0.01 mL of blood sample was pipetted into the prepared test tubes, followed by incubation for 30 min. The blood samples were mixed with a glass rod to obtain a homogeneous cell suspension. The tubes were then centrifuged (Centrifuge 5702, Eppendorf, Hamburg, Germany) for 10 min at 2200 rpm at 4 °C. The supernatant was then measured spectrophotometrically (Libra S22, Biochrom, Cambridge, UK) at 540 nm, with the first tube (0.9% NaCl) used as a blank.

### 4.7. Analysis of Essential and Toxic Metals by Inductively Coupled Plasma Mass Spectrometry (ICP-MS)

#### 4.7.1. Standards and Chemicals

For calibration of inductively coupled plasma mass spectrometer (ICP-MS), certified multi-element standards with 99.99% purity for all elements were used (concentration of 10 mg/L, composed of aluminum, cadmium, copper, iron, manganese, lead, selenium, and zinc; Environmental Calibration Standard, Agilent Technologies, Santa Clara, CA, USA). Stock solutions for ICP-MS analysis were prepared by dissolving the multi-element standard mixture solution with ultrapure water (18.2 MΩ/cm resistivity) obtained from system Milli-Q Advantage 10 V (Millipore Corporation Merck, Darmstadt, Germany). Stock solutions were diluted with 5% (*v/v*) HNO_3_ (Merck, Darmstadt, Germany) by serial dilution in working solutions and kept at room temperature until further use. Prior to use, all glassware and plastic were cleaned by soaking in diluted HNO_3_ (5%, *v/v*) and rinsed with ultrapure water. Ultrapure-grade carrier argon (Ar, 99.9995% pure) was supplied by UTP d.o.o. (Pula, Croatia).

#### 4.7.2. Sampling and Sample Preparation for ICP-MS

Liver, kidney, spleen, and lung tissue samples were isolated and stored in polypropylene tubes with stoppers at −80 °C until ICP-MS analysis. During the isolation and storage of the samples, measures were taken to prevent contamination between the treated groups. A total of 0.5 g of liver, kidney, spleen, and lung tissue samples were weighed into Teflon dish with the addition of 2.5 mL HNO_3_ (65% *v/v*), 2 mL H_2_O, and 1 mL H_2_O_2_ (30% *v/v*, Carlo Erba reagents, Rodano, Italia). Microwave digestion was carried out on a high-pressure microwave oven (Multiwave 3000, Anton Paar, Graz, Austria), which had 16 dishes in which microwave digestion of samples was carried out. The digestion program was carried out in three steps: the first step at 500 W for 4 min, the second step at 1000 W for 20 min, and the third step at 1200 W for 30 min. After digestion and cooling, the samples were quantitatively transferred into volumetric flasks of 50 mL and made up to the mark with ultra-pure water. The mix of the internal standard (ISTD) solution containing In, Bi, and Sc was added online using the standard ISTD mixing tee-connector. The samples were processed in triplicate.

Detection limits were determined as the concentration corresponding to three times the standard deviation of 10 blanks. All samples were run in batches that included blanks, a standard calibration curve, two spiked specimens, and one duplicate. All metal concentrations were determined on a wet weight basis as mg/kg. The quality of data was checked by the analysis of recovery rate using certified reference materials: bovine liver (BCR 185R, IRMM, Geel, Belgium) and DORM-4 Fish protein certified reference material for trace metals (National Research Council Canada, Ottawa, ON, Canada).

#### 4.7.3. Metal Analysis

The concentration of essential and toxic metals in the liver, kidney, spleen, and lungs was measured using an inductively coupled plasma instrument with the mass detector Agilent ICP-MS system Model 7900 (Agilent, Palo Alto, CA, USA). The ICP-MS working parameters and conditions are already described in paper by Bilandžić et al. [79]. The sample introduction system consisted of a quartz cyclonic spray chamber and a Meinhard^®^ nebulizer connected to the peristaltic pump of the spectrometer with Tygon^®^ tubes. The ICP-MS was operated with a platinum sampler and skimmer cones. The peristaltic pump of the ICP-MS was set at 0.40 rps. High-purity argon (99.999%, White Martins, Rio de Janeiro, Brazil) was used throughout. Optimization of the ICP-MS conditions was achieved by adjusting the torch position and tuning for reduced oxide and doubly charged ion formation with a standard tuning solution containing Li, Y, Ce, and Tl in 2% HNO_3_.

### 4.8. Measurement of Hepcidin and Ferritin Levels in Serum

Hepcidin and ferritin levels were measured using commercially available kits and performed according to the manufacturer’s instructions. The level of hepcidin in the serum was measured using an ELISA kit for hepcidin (Enzyme-linked Immunosorbent Assay Kit for Hepcidin) manufactured by Cloud-Clone Corp., Houston, TX, USA, while the level of ferritin in the serum was measured using the Enzyme-linked Immunosorbent Assay Kit for Ferritin manufactured by Cloud-Clone Corp., Houston, TX, USA. Serum samples were diluted twice with phosphate-buffered saline (Lonza, Basel, Switzerland). The samples were measured in duplicate, and the dilution factor was considered in the final calculation of the concentration. Absorbance values were read at 450 nm on Ao Absorbance Microplate Reader (Azure Biosystems, Dublin, CA, USA). From the standard curve of the dependence of the logarithmic concentration of hepcidin on the absorbance, the equation of the direction was determined, and the concentrations of hepcidin in the serum were expressed in ng/mL, while the concentration of ferritin was calculated from the standard curve of the dependence of the absorbance on the concentration of the standard ferritin solution, where the equation of the direction was determined, and the concentrations serum ferritin were also expressed in ng/mL. The samples were made in duplicate, and the dilution factor was considered in the final calculation of the concentration.

### 4.9. Determination of the Concentration of Matrix Metalloproteinase 2 (MMP-2) and 9 (MMP-9)

Matrix metalloproteinase 2 (MMP-2) and 9 (MMP-9) concentrations were determined using the MMP-2 Rat ELISA Kit and MMP-9 Rat ELISA Kit manufactured by Chongqing Biospes Co., Ltd., Biospes, Chongqing, China, according to the manufacturer’s instructions in serum diluted 10× with sample dilution kit buffer. The intensity of the resulting staining is proportional to the concentration of rat’s MMP-2 or MMP-9, and the absorbance values were read at 450 nm on the Ao Absorbance Microplate Reader (Azure Biosystems, Dublin, CA, USA). From the standard curve of dependence of absorbance on the concentration of the MMP-2 or MMP-9 standard solution, the direction equation was determined, and the concentrations of MMP-2 or MMP-9 in the serum were expressed in ng/mL. The samples were made in duplicate, and the dilution factor was considered in the final calculation of the concentration.

### 4.10. Statistical Analysis

Statistical analysis was performed using the STATISTICA 14 program (StatSoft, Tulsa, OK, USA). All data were presented as mean ± standard error (mean ± SE). The data were analyzed using the Kruskal–Wallis nonparametric test and were considered significant at the level of *p* < 0.05. Further analysis of the differences between the groups was made by multiple comparisons of the mean values of all groups. Some data are represented using GraphPad Prism 7 software (GraphPad Software, Inc., La Jolla, CA, USA).

## Figures and Tables

**Table 1:** Iron concentration in the peripheral organs of rats exposed to chronic administration of inhalation anesthetics and iron dextran and their combination.

Groups ^a^	Iron (mg/kg) (Mean ± SE)			
	Liver	Kidney	Lungs	Spleen
Cont.	409.78 ± 6.86	68.35 ± 1.18	70.19 ± 3.17	1651.57 ± 44.18
Sevo.	761.72 ± 27.82	75.89 ± 0.38	83.23 ± 1.80	2712.07 ± 32.59
Iso.	262.63 ± 2.97	85.31 ± 3.10	116.56 ± 5.64	1312.69 ± 38.23
Fe-dex.	7797.16 ± 88.12 *^∆∆^	173.87 ± 2.74 *	218.00 ± 8.37 *	11,069.39 ± 161.33 *^∆∆^
Fe-dex. + Sevo.	10,945.52 ± 11.14 **^■∆∆∆^	218.23 ± 6.07 **	314.09 ± 4.68 **^■^	13,371.78 ± 100.13 **^■∆∆∆^
Fe-dex. + Iso.	3813.77 ± 3.94 ^∆^	346.43 ± 4.89 ***^■■∆^	397.20 ± 2.28 ***^■■∆^	6440.82 ± 126.14 ^∆^

^a^ Rats (n = 10 per group) were treated with *ip* injection of iron dextran (50 mg/kg), exposed to 2.4 vol % Sevoflurane inhalation and 1.3 vol % Isoflurane inhalation, and exposed to a combination thereof where inhaled anesthetics were given 2 h after *ip* injection of iron dextran. Animals were treated every other day for 28 days. The results are expressed as the mean value of each experimental group ± SE. * Statistically significant compared to the control group (* *p* < 0.05; ** *p* < 0.01; *** *p* < 0.001); ^■^ statistically significant compared to the Sevoflurane group (^■^ *p* < 0.05; ^■■^ *p* < 0.01); ^∆^ statistically significant compared to the Isoflurane group (^∆^ *p* < 0.05; ^∆∆^ *p* < 0.01; ^∆∆∆^ *p* < 0.001). Abbreviations: *ip*, intraperitoneal; SE, standard error; Cont., control group; Sevo., Sevoflurane group; Iso., Isoflurane group; Fe-dex., iron dextran group; Fe-dex. + Sevo., group treated with iron dextran and Sevoflurane; Fe-dex. + Iso., group treated with iron dextran and Isoflurane.

**Table 2:** Concentrations of essential metals in the peripheral organs of rats exposed to chronic administration of inhalation anesthetics and iron dextran and their combination.

Groups ^a^	Essential Metals (mg/kg)—Mean ± SE Liver			
	Manganese	Copper	Zinc	Selenium
Cont.	2.51 ± 0.04	5.03 ± 0.19	16.13 ± 0.38	1.46 ± 0.03
Sevo.	2.78 ± 0.09	4.88 ± 0.05	14.26 ± 0.19	1.40 ± 0.02
Iso.	2.93 ± 0.08	4.65 ± 0.09 ^∇∇^	31.90 ± 1.15 **^■■■^	1.24 ± 0.07
Fe-dex.	5.17 ± 0.06 *	5.07 ± 0.04	28.02 ± 0.16 ^■^	1.35 ± 0.02
Fe-dex. + Sevo.	5.68 ± 0.18 **	7.14 ± 0.12	29.91 ± 0.63 *^■■^	1.32 ± 0.02
Fe-dex. + Iso.	0.27 ± 0.00 ^###∇∇∇^	1.95 ± 0.03 *^#∇∇∇^	18.27 ± 0.13	0.41 ± 0.01 ***^■^
Groups ^a^	Kidney			
	Manganese	Copper	Zinc	Selenium
Cont.	0.96 ± 0.01	12.65 ± 0.19	12.32 ± 0.03	1.40 ± 0.02
Sevo.	0.91 ± 0.02	8.17 ± 0.54	11.11 ± 0.27	1.42 ± 0.03
Iso.	1.02 ± 0.04	7.79 ± 0.31	22.08 ± 0.12 **^■■■♦^	1.34 ± 0.02
Fe-dex.	0.93 ± 0.03	6.82 ± 0.09 **	20.08 ± 0.37 ^■^	1.34 ± 0.02
Fe-dex. + Sevo.	1.00 ± 0.02	7.21 ± 0.06	20.98 ± 0.20 ^■■^	1.18 ± 0.00 *^■^
Fe-dex. + Iso.	0.51 ± 0.02 *^∆∆∇∇^	2.93 ± 0.03 ***^■^	13.27 ± 0.52	0.15 ± 0.00 **^■■■#^
Groups ^a^	Lungs			
	Manganese	Copper	Zinc	Selenium
Cont.	0.22 ± 0.00	2.15 ± 0.06	10.04 ± 0.12	0.42 ± 0.01
Sevo.	0.21 ± 0.00	1.86 ± 0.02 ^∇∇^	9.20 ± 0.15	0.39 ± 0.01
Iso.	0.29 ± 0.01	2.04 ± 0.10	17.87 ± 0.45 *^■■■^	0.45 ± 0.01
Fe-dex.	0.23 ± 0.01	1.94 ± 0.01	16.58 ± 0.03 ^■^	0.39 ± 0.00
Fe-dex. + Sevo.	0.31 ± 0.00 ^■^	3.23 ± 0.05	17.67 ± 0.09 **^■■■^	0.36 ± 0.00 *^∆∆^
Fe-dex. + Iso.	2.47 ± 0.09 ***^■■■#^	1.25 ± 0.06 **^∇∇∇^	15.57 ± 0.15	0.56 ± 0.02 ^■##∇∇∇^
Groups ^a^	Spleen			
	Manganese	Copper	Zinc	Selenium
Cont.	0.28 ± 0.01	1.59 ± 0.02	10.42 ± 0.08	0.60 ± 0.01
Sevo.	0.58 ± 0.02	1.50 ± 0.03	9.83 ± 0.04	0.61 ± 0.01
Iso.	0.17 ± 0.01	1.20 ± 0.02	16.49 ± 0.46	0.45 ± 0.01
Fe-dex.	2.56 ± 0.04 *^∆∆^	1.58 ± 0.02	18.50 ± 0.25 ^■^	0.64 ± 0.02
Fe-dex. + Sevo.	3.22 ± 0.01 **^■∆∆∆^	2.10 ± 0.30 ^∆∆^	21.38 ± 0.50 **^■■■^	0.61 ± 0.00
Fe-dex. + Iso.	1.05 ± 0.00 ^∆^	5.71 ± 0.52 ^■■∆∆∆^	21.31 ± 0.15 *^■■■^	1.38 ± 0.01 ^∆∆∆^

^a^ Rats (n = 10 per group) were treated with *ip* injection of iron dextran (50 mg/kg), exposed to 2.4 vol % Sevoflurane inhalation and 1.3 vol % Isoflurane inhalation, and exposed to a combination thereof where inhaled anesthetics were given 2 h after *ip* injection of iron dextran. Animals were treated every other day for 28 days. The results are expressed as the mean value of each experimental group ± SE. * Statistically significant compared to the control group (* *p* < 0.05; ** *p* < 0.01; *** *p* < 0.001); ^■^ statistically significant compared to the Sevoflurane group (^■^ *p* < 0.05; ^■■^ *p* < 0.01; ^■■■^ *p* < 0.001); ^∆^ statistically significant compared to the Isoflurane group (^∆^ *p* < 0.05; ^∆∆^ *p* < 0.01; ^∆∆∆^ *p* < 0.001); ^#^ statistically significant compared to the iron dextran-treated group (^#^ *p* < 0.05; ^##^ *p* < 0.01; ^###^ *p* < 0.001); ^∇^ statistically significant compared to the Fe-dex. + Sevo.-treated group (^∇∇^ *p* < 0.05; ^∇∇∇^ *p* < 0.001); ^♦^ statistically significant compared to the Fe-dex. + Iso.-treated group (^♦^ *p* < 0.05). Abbreviations: *ip*, intraperitoneal; SE, standard error; Cont., control group; Sevo., Sevoflurane group; Iso., Isoflurane group; Fe-dex., iron dextran group; Fe-dex. + Sevo., group treated with iron dextran and Sevoflurane; Fe-dex. + Iso., group treated with iron dextran and Isoflurane.

**Table 3:** Aluminum concentration in the peripheral organs of rats exposed to chronic administration of inhalation anesthetics and iron dextran and their combination.

Groups ^a^	Aluminum (mg/kg) (Mean ± SE)			
	Liver	Kidney	Lungs	Spleen
Cont.	0.55 ± 0.03	0.44 ± 0.02	0.53 ± 0.01	0.61 ± 0.01
Sevo.	0.51 ± 0.01	0.42 ± 0.02	0.55 ± 0.02	1.20 ± 0.01 ^∆∆^
Iso.	0.43 ± 0.02	0.57 ± 0.03	2.53 ± 0.02	0.50 ± 0.02
Fe-dex.	3.67 ± 0.06 ^∆∆^	0.51 ± 0.01	0.58 ± 0.02	4.73 ± 0.06 *^∆∆∆♦♦^
Fe-dex. + Sevo.	7.45 ± 0.26 *^■■∆∆∆^	2.95 ± 0.03 *^■■♦♦♦^	3.62 ± 0.12 **^■^	0.74 ± 0.07
Fe-dex. + Iso.	0.63 ± 0.02	0.19 ± 0.01 ^∆∆#^	3.86 ± 0.09 ***^■■#^	0.63 ± 0.12

^a^ Rats (n = 10 per group) were treated with *ip* injection of iron dextran (50 mg/kg), exposed to 2.4 vol % Sevoflurane inhalation and 1.3 vol % Isoflurane inhalation, and exposed to a combination thereof where inhaled anesthetics were given 2 h after *ip* injection of iron dextran. Animals were treated every other day for 28 days. The results are expressed as the mean value of each experimental group ± SE. * Statistically significant compared to the control group (* *p* < 0.05; ** *p* < 0.01; *** *p* < 0.001); ^■^ statistically significant compared to the Sevoflurane group (^■^ *p* < 0.05; ^■■^ *p* < 0.01); ^∆^ statistically significant compared to the Isoflurane group (^∆∆^ *p* < 0.01; ^∆∆∆^ *p* < 0.001); ^#^ statistically significant compared to the iron dextran-treated group (^#^ *p* < 0.05); ^♦^ statistically significant compared to the Fe-dex. + Iso.-treated group (^♦♦^ *p* < 0.01; ^♦♦♦^ *p* < 0.001). Abbreviations: *ip*, intraperitoneal; SE, standard error; Cont., control group; Sevo., Sevoflurane group; Iso., Isoflurane group; Fe-dex., iron dextran group; Fe-dex. + Sevo., group treated with iron dextran and Sevoflurane; Fe-dex. + Iso., group treated with iron dextran and Isoflurane.

**Table 4:** Toxic metal concentrations in the peripheral organs of rats exposed to chronic administration of inhalation anesthetics and iron dextran and their combination.

Groups ^a^	Toxic Metals (µg/kg)—Mean ± SE			
	Liver		Kidney	
	Cadmium	Lead	Cadmium	Lead
Cont.	12.32 ± 0.44	1.94 ± 0.07	34.04 ± 0.12	3.70 ± 0.38
Sevo.	16.75 ± 0.31	3.19 ± 0.39	38.90 ± 0.99	3.60 ± 0.32
Iso.	8.90 ± 0.83 ^■■^	1.95 ± 0.10	16.69 ± 0.59 ^■■■###♦^	2.87 ± 0.24
Fe-dex.	13.67 ± 0.85	27.11 ± 0.86 *^∆^	39.46 ± 1.76	1.23 ± 0.08
Fe-dex. + Sevo.	14.71 ± 0.86	250.95 ± 9.83 ***^∆∆∆♦^	28.52 ± 1.18 ^■#^	117.54 ± 3.60 ^##^
Fe-dex. + Iso.	0.41 ± 0.03 ^■■■∇∇^	2.31 ± 0.31	35.44 ± 0.23	0.69 ± 0.04 *^■∇∇∇^
Groups ^a^	Lungs		Spleen	
	Cadmium	Lead	Cadmium	Lead
Cont.	1.22 ± 0.07	1.34 ± 0.05	2.26 ± 0.20	2.61 ± 0.23
Sevo.	1.27 ± 0.08	2.87 ± 0.29	2.59 ± 0.15	10.07 ± 0.16
Iso.	0.70 ± 0.03 **^■■^	2.35 ± 0.30	0.64 ± 0.03	0.71 ± 0.07
Fe-dex.	0.83 ± 0.03	0.00 ± 0.00	1.84 ± 0.15	32.80 ± 0.34 **^∆∆∆^
Fe-dex. + Sevo.	1.48 ± 0.16 ^∆∆∆#^	164.07 ± 8.35 **^###^	1.84 ± 0.25	10.18 ± 1.86
Fe-dex. + Iso.	0.89 ± 0.04	30.30 ± 0.72 *^##^	29.13 ± 2.12 ^∆∆∆#∇^	15.48 ± 0.19 ^∆∆^

^a^ Rats (n = 10 per group) were treated with *ip* injection of iron dextran (50 mg/kg), exposed to 2.4 vol % Sevoflurane inhalation and 1.3 vol % Isoflurane inhalation, and exposed to a combination thereof where inhaled anesthetics were given 2 h after *ip* injection of iron dextran. Animals were treated every other day for 28 days. The results are expressed as the mean value of each experimental group ± SE. * Statistically significant compared to the control group (* *p* < 0.05; ** *p* < 0.01; *** *p* < 0.001); ^■^ statistically significant compared to the Sevoflurane group (^■^ *p* < 0.05; ^■■^ *p* < 0.01; ^■■■^ *p* < 0.001); ^∆^ statistically significant compared to the Isoflurane group (^∆^ *p* < 0.05; ^∆∆^ *p* < 0.01; ^∆∆∆^ *p* < 0.001); ^#^ statistically significant compared to the iron dextran-treated group (^#^ *p* < 0.05; ^##^ *p* < 0.01; ^###^ *p* < 0.001); ^∇^ statistically significant compared to the Fe-dex. + Sevo.-treated group (^∇^ *p* < 0.05; ^∇∇^ *p* < 0.01; ^∇∇∇^ *p* < 0.001); ^♦^ statistically significant compared to the Fe-dex. + Iso.-treated group (^♦^ *p* < 0.05). Abbreviations: *ip*, intraperitoneal; SE, standard error; Cont., control group; Sevo., Sevoflurane group; Iso., Isoflurane group; Fe-dex., iron dextran group; Fe-dex. + Sevo., group treated with iron dextran and Sevoflurane; Fe-dex. + Iso., group treated with iron dextran and Isoflurane.

## Data Availability

The original contributions generated for this study are included in the article; further inquiries can be directed to the corresponding author.
